# Cerebral Metastases from Malignant Melanoma: Current Treatment Strategies, Advances in Novel Therapeutics and Future Directions

**DOI:** 10.3390/cancers2020364

**Published:** 2010-04-06

**Authors:** Timothy L. Siu, Suyun Huang

**Affiliations:** Department of Neurosurgery, The University of Texas MD Anderson Cancer Center, Houston, TX 77030, USA; E-Mail: siloti@tpg.com.au (T.S.)

**Keywords:** cerebral metastasis, melanoma, surgery, radiotherapy, drug therapy, STAT3 transcription factor

## Abstract

Of all primary cancers in humans, melanoma has the highest propensity to metastasize to the brain. The prognosis of patients with this disease is extremely poor. Due to its radioresistance and poor response to existing chemotherapeutic regimes, no treatment options other than surgical extirpation, when feasible, have been shown to be effective. An understanding of the underlying tumor biology therefore remains the cornerstone of offering new hope in the treatment. In this review, we comment on the current treatment strategies for melanoma brain metastases and summarize some recent experimental findings from our laboratory with potential for the development of target specific antitumor therapies.

## 1. Introduction

Melanoma is one of the deadliest forms of cancer known to humans. Unlike other common cancer types such as breast and lung cancers, its incidence has been steadily rising over the decades. In 2009, melanoma was the sixth most common cancer in the US, with more than 68,000 cases diagnosed annually leading to about 8,600 deaths each year [1]. Of all primary tumors, it has the highest propensity to metastasize to the brain—up to 75% of all patients who died from melanoma harbor brain metastases and in 50% of these patients, brain metastasis is the cause of death [2,3,4,5]. Despite such an alarming trend, the clinical outcome of patients with melanoma brain metastases has been dismal, with survival averaging less than six months, notwithstanding standard combined treatment with surgery and radiation therapy [6,7]. In order to devise more effective treatment regimes, research into the underlying pathomechanisms of melanoma brain metastases currently carries the only hope for the development of novel systemic therapies against this deadly disease. In this review, we will summarize the key clinical characteristics of melanoma brain metastases, the current therapeutic options and some recent discoveries on the pathogenesis of melanoma brain metastasis from our laboratory and their implications for the development of new therapeutics.

## 2. Clinicopathological Characteristics

**Figure 1:**
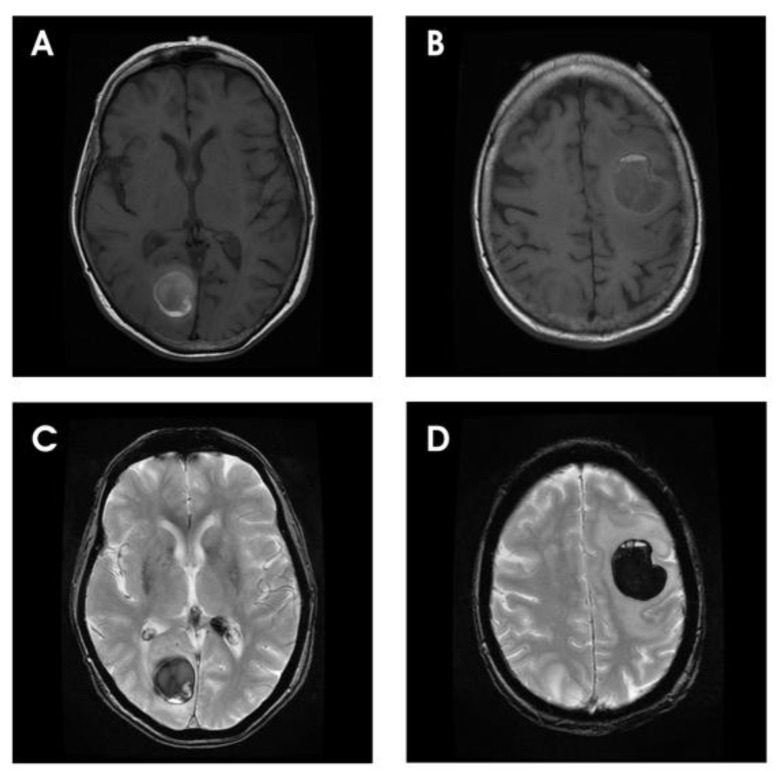
Axial MRI demonstrating multiple hemorrhagic lesions in a patient with metastatic melanoma. (A–B) T1 weighted images show two circumscribed lesions in the left frontal and the right occipital lobes. The mixed signal intensity within the lesion suggests the presence of hemorrhages of various chronicity. (C–D) T2* weighted sequence confirms the presence of hemorrhages as indicated by the areas of hypointensity. Additional hemorrhagic lesions are also shown in the left lateral ventricle and in the posterior third ventricle.

The neurological symptoms and signs of melanoma brain metastases, caused by either the primary local mass effect of the tumor on adjacent brain tissue or secondary effects from raised intracranial pressure or impediment of cerebrospinal fluid (CSF) circulation, are indistinguishable from those of any other intracranial space-occupying lesions. However, characteristically, hemorrhagic transformation occurs frequently in melanoma metastases and consequently patients with melanoma metastases often present acutely with headaches, focal neurological deficits and seizures, likened by some authors to “tumor TIA” (Figure 1) [8]. With increasing availability of sophisticated modern imaging, such as MRI and PET/CT, an increasing number of asymptomatic patients are also diagnosed incidentally or during disease staging [6].

## 3. Radiotherapy

Whole brain radiotherapy (WBRT) has been the cornerstone of treatment of metastatic brain tumors for over half a century. Historically the median survival of patients harboring brain metastases with supportive care alone is 12-months and WBRT extends the survival to 36-months [9]. Nevertheless, of all primary tumor types, melanoma is considered to be one of the most resistant to this mode of treatment [10]. In addition, as melanoma is largely underrepresented in the current established WBRT literature [11,12,13], the role of WBRT for the treatment of melanoma brain metastasis remains unproven. In fact based on available retrospective data specific to examining the treatment outcome of melanoma brain metastases [7,14,15,16,17], the overall reported median survival of patients even with WBRT ranges only from 26–months and these figures beg the question on the benefits of the routine application of WBRT for melanoma metastases. 

On the other hand, although WBRT is generally considered safe and noninvasive, some emerging evidence has suggested that important drawbacks, including fatigue, drowsiness and neurocognitive sequelae [9], could greatly impair the quality of life of patients treated with WBRT. In a recent randomized controlled trial, using specific neuropsychological assessment tools, it was established that patients receiving WBRT suffered a significantly greater decline in memory and learning compared with patients receiving localized radiation with stereotactic radiosurgery (SRS) alone [18]. Taking these into consideration, when contemplating WBRT as a treatment option, the risk and benefit of WBRT should therefore be carefully weighed in each individual patient and preference should be given to other more effective treatment alternatives when available.

## 4. Surgery

Metastatic brain tumors characteristically form circumscribed and rounded masses, rendering them highly amenable to surgical extirpation and the delivery of a “local cure” following complete tumor excision. This has particularly been the case for patients harboring only a single metastasis as the results from three landmark surgical randomized controlled trials collectively demonstrate that patients survive longer with lower recurrence after combined treatment with surgical resection and WBRT than WBRT alone [19,20,21,22]. For patients harboring multiple brain metastases, however, the survival benefits derivable from surgery are less clear, as data suggest that patient outcome is tied in more closely with the systemic tumor burden than the central nervous system (CNS) disease [23]. In view of these, as patients with cerebral metastases represent a heterogeneous group of patients with less than 30% having only a single metastasis, a custom approach is therefore required to tailor the utilization of surgery to the clinical condition of each individual patient. 

**Figure 2:**
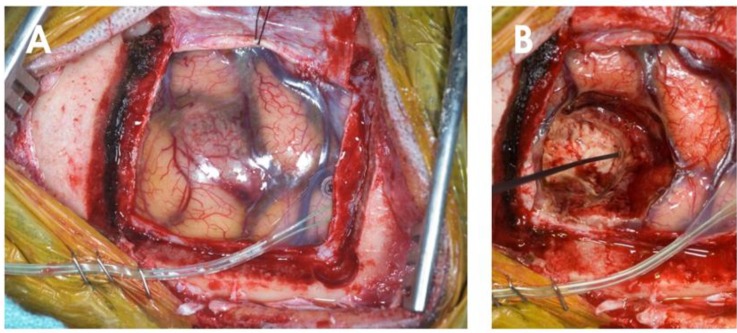
Intraoperative photographs showing the application of electrophysiological recording to ensure removal of the left frontal tumor as demonstrated in Figure 1 without injuring the adjacent motor tract. (A) A paramedian craniotomy was created over the left premotor region. A dural leaf was reflected medially exposing the tumor and the adjacent cortical gyri. Cortical mapping was performed with somatosensory evoked potential recording from a multielectrode array placed posteriorly across the central sulcus. A characteristic phase reversal can differentiate signals originating from the primary motor cortex from those from the primary sensory cortex. (B) A fine tipped stimulating electrode was applied to stimulate the tumor cavity to ascertain the location of the descending motor tract.

It is undeniable that surgery can play an indispensable palliative role in alleviating neurological symptoms from the local mass effect of the tumor even for patients with advanced disseminating disease. Currently no other treatment modality can effect immediate reduction of tumor mass effect, neural decompression, and restoration of CSF flow more efficiently than surgery. This is particularly the case when the tumor is located in the posterior fossa when expedient surgical resection could mean life or death in preventing fatal brainstem compression or herniation. With modern neurosurgical armamentarium including neuronagvigation, intraoperative imaging and functional mapping, surgery for brain metastases can be performed in unprecedentedly precise and controlled manner with minimal morbidity and mortality (Figure 2) [24]. It is therefore of paramount importance that all patients with cerebral metastases are assessed as candidates for surgical resection irrespective of their overall cancer status or the multiplicity of their CNS disease.

One significant, but often unrecognized technical issue when performing surgery on cerebral metastases is the notion of en bloc *versus* piecemeal resection in minimizing intraoperative tumor spillage. Because brain metastases are circumscribed tumors, it has been postulated that the violation of tumor capsule and perturbation of tumor content during piecemeal resection could lead to dissemination of neoplastic substrates into the neuraxis whilst en bloc resection along a gliotic plane in the brain parenchyma could preserve the natural biological containment of the tumor cells [25,26]. This speculation has been supported by some retrospective data in showing a higher rate of leptomeningeal disease observed in patients undergoing piecemeal metastatic tumor resection than patients having en bloc resection [25,26]. For metastatic melanoma, however, as tumor hemorrhage often occurs, the importance of tumor seeding due to operative maneuvers may be played down by the fact that the pressure generated during the ictus of hemorrhage would inevitably rupture the tumor capsule, seeding a myriad of microscopic tumor foci into the locoregional milieu. Nevertheless it would still make good oncologic sense and be prudent, when feasible, to perform en bloc resection, particularly allowing for generous margin to eradicate local hemorrhagic seeding for melanoma to improve local disease control, reserving piecemeal resection in cases when the tumor is too large in size or when adjacent brain eloquence precludes safe en bloc resection. Further prospective data will help clarify these issues.

## 5. Stereotactic Radiosurgery

SRS delivers a single large dose of focused radiation to destroy lesions localized by stereotaxy. It minimizes radiation exposure to normal brain parenchyma through crossfiring from many directions, which results in rapid radiation falloff in the surrounding tissue. Importantly, the tumoricidal mechanism of SRS, believed to mediate through changes in tumor vasculature, is different from WBRT and hence tumors traditionally regarded as “radioresistant” such as melanoma, renal cell carcinoma and sarcoma has exhibited susceptibility to SRS [27]. As a primary treatment modality, SRS has been shown to be effective for melanoma metastases [28]. Multiple retrospective series have indicated a median survival of 61–0 months following treatment of SRS for patients with either single or multiple brain metastases [29,30,31,32]. These results compare favorably with results obtained from numerous surgical series for melanoma brain metastases of 51–0 months [7,33,34,35]. Although such retrospective data comparison is fraught with potential pitfalls relating to inherent selection and follow-up biases and the fundamental difference in the patient population studied, attempts to conduct prospective randomized studies to compare the role of surgery *vs.* SRS in the management of cerebral metastases in general has not been successful due to significant obstacles in patient accrual [36]. Current practice therefore has to rely on judicious evaluation of available retrospective data.

SRS offers a few advantages over conventional surgery. It can treat inaccessible tumor without the increased risks of surgical resection, especially when eloquent brain has to be transgressed to reach the lesion. It is also less invasive, requires shorter hospital stays because only a single-fraction of radiation is given, and can be offered to patients who have major cardiac, pulmonary, renal, or hematologic diseases and cannot tolerate surgery. On the other hand some major drawbacks of SRS include the restriction in treating only small tumors (generally <3 cm) and the lack of immediate treatment effect. The former is due to the fact that there exists a limit in conformity that can be achieved for large tumor volume (>3 cm) and consequently treatment of such tumors with SRS could result in an integral radiation dose to the surrounding brain parenchyma of an unacceptably high level [37]. The latter occurs because the tumoricidal effect of SRS relies on the disruption of normal cellular activity and proliferation and cell death which evolves gradually over a period of weeks to months. In light of these, a collaborative approach between neurosurgeons and radiation oncologists is therefore vital in providing a complementary utilization of both modalities. For instance, a patient harboring multiple small deep-seated tumors with a dominant large symptomatic lesion would benefit most from a combined treatment with SRS for the smaller lesions and surgery for the dominant symptomatic lesion. Such an approach conceivably provides the best local disease control and for melanoma patients with increased propensity for developing multiple brain metastases is particularly desirable as alternative, global therapy with WBRT often lacks treatment efficacy as discussed above [38].

In the past, WBRT has been offered as a standard adjuvant therapy following local treatment with surgery or SRS. This is based on evidence from randomized controlled studies that demonstrate tumor relapse rates were higher when WBRT was forgone (although no difference in survival rates was observed with or without WBRT) [39,40]. However, as melanoma is again largely underrepresented in these studies, the true efficacy of WBRT in preventing melanoma metastasis relapse cannot be conclusively established based on current evidence. On the other hand, a recent randomized study from MD Anderson Cancer Center which demonstrates patients with cerebral metastases having more significant deterioration in their neurocognitve functions after adjuvant WBRT than their no-WBRT counterparts affirms the speculation concerning the harmful neurological sequelae of WBRT [18].

In view of these, some authors have suggested that instead of applying WBRT, SRS could be considered as a boost treatment to the tumor resection cavity for the purpose of minimizing local tumor recurrence, especially for patients with “radioresistant” tumors like melanoma [41,42]. With this premise, at MD Anderson Cancer Center, a phase III randomized controlled trial was opened in August 2009 to evaluate the efficacy of postoperative SRS on the resection bed of cerebral metastases in reducing the risk of local tumor recurrence at six months. This trial, expected to complete in 2014, will provide important data to clarify the proper use of this adjunctive treatment.

## 6. Chemotherapy

The results of the use of chemotherapy for melanoma brain metastases have largely been disappointing. Trials of conventional agents such as cisplatin, etoposide and carmustine have all failed to demonstrate any significant treatment response [43,44]. The use of newer agents such as temozolomide, which is marked by its excellent side effect profile and CNS penetration, with or without concurrent WBRT, has also been unsuccessful in producing any significant improvement, with an overall reported response rate of less than 10% [45,46]. The current evidence therefore does not support the routine use of existing chemotherapeutic agents for the treatment of melanoma cerebral metastases.

## 7. Targeted Molecular Therapy

The mechanisms underlying the metastatic process consist of a complex cascade of events, involving tumor cell mediated invasion at the primary site, migration and survival in the host vasculature, and extravasation and colonization at the distant microenvironment. Each of these steps are orchestrated by an integral interplay of intracellular and extracellular signaling involving various oncogenes, tumor suppressor genes, metastasis suppressor genes, and growth factors and their receptors. Importantly, altered expression of several signaling molecules identified in human melanoma cell lines has been implicated in melanoma brain metastasis *in vivo*, providing potential targets for the development of new therapeutics. Of note, one critical step in tumor cell invasion and migration modulated by these targeted molecules involves the degradation of extracellular matrix (ECM) and endothelial cells underpinning the blood-brain barrier (BBB) [47,48]. The fibrinolytic system, through the activation of plasmin and matrix metalloproteinases (MMPs) [49], and the growth factors, neurotrophins, mediating in part via the activation of the degradative enzymes heparanase [47], are two of the mechanistic pathways shown to be responsible for the breakdown of the major protein and glycosaminoglycans components of the ECM respectively involved in the development of melanoma brain metastasis. A monoclonal antibody targeting a specific melanoma cell surface antigen, melanotransferrin, that exhibits excitatory binding to plasminogen, is one recent example of applying such mechanistic insights in successfully preventing the development of brain metastasis in a laboratory model [49].

Apart from these fledgling molecular targets, a ubiquitous signal transduction pathway regulator, the signal transducer and activator of transcription 3 (STAT3) protein, has recently been shown in our laboratory to also play a critical role in melanoma induced brain metastases [50]. Unlike the above candidates, STAT3 serves multiple prometastatic roles through regulation of downstream signal transduction pathways involving tumor cell apoptosis, migration, evasion of immune surveillance and angiogenesis central to the development of metastasis [51]. A mechanistic understanding thereof opens novel therapeutic avenues for not only treatment but prevention of brain metastases of melanoma, which may prove to be more effective than the currently available therapies such as the anti-vascular endothelial growth factor (VEGF) antibody, bevacizumab, or experimental treatment targeting the fibrinolytic system, in tackling only one aspect of metastases. 

By studying human melanoma cell lines obtained from surgical specimens *in vitro*, we found that STAT3 was constitutively activated at a substantially higher level in brain melanoma tissues [52]. Fittingly, by transfecting melanoma cells with a constitutively activated mutant form of STAT3, we noted that nude mice injected with these cells had a dramatic increase in the incidence of rapidly progressing brain metastases and shortened survival when compared with wild type control [50]. In sharp contrast, when nude mice were injected with STAT3DN transfected cells, a mutant form with reduced tyrosine phosphorylation resulting in inhibition of endogenous STAT3 activation, a very low incidence or no brain metastatic lesions were observed [50]. Consistent with these findings, the study of JAK2 and SOCS-1 protein that regulate STAT3 expression yielded corroborative evidence supporting the role of SOCS-1/JAK2/STAT3 signaling pathway in the development of melanoma brain metastasis [53].

The SOCS-1/JAK2/STAT3 signaling pathway affects the development of cerebral metastases through its regulation of the gene transcription of downstream signaling transduction molecules. Basic fibroblast growth factor (bFGF), VEGF and MMP-2 are some of these important molecules underlying tumor angiogenesis and growth. By evaluating the expression of bFGF, VEGF and MMP-2 in metastatic tumors derived from melanoma cell lines with STAT3 activation altered, we established that STAT3 activation has, as expected, a direct correlation with increased expression of these angiogenic molecules and corresponding increases in tumor microvessel density as demonstrated on immunohistochemistry [50]. Specifically as MMP-2 serves to degrade ECM and basement membranes underlying tumor cell invasion and migration, we showed by using an invasion assay *in vitro* that STAT3 downregulated tumor cell lines exhibit significant decreased invasion capability when compared with control [50]. These observations, taken together, demonstrate the multiple mechanistic roles predicted of STAT3 on melanoma brain metastasis and the therapeutic potential of targeting this signaling pathway for preventing tumor progression.

## 8. Conclusions and Future Directions

The management of metastatic brain tumors from melanoma remains a formidable challenge—their predilection to hemorrhage and their resistance to conventional radiotherapy defy the traditional role of surgery and WBRT in attaining local and distant disease control; the underrepresentation of melanoma in prospective randomized studies regarding cerebral metastases undermines the application of current standard treatment based on available evidence. While refinement in existing treatment options, such as advances in operative techniques in achieving radical yet safe tumor resection and the application of adjuvant SRS to tumor bed to improve local disease control, may translate to improved survival, a rational approach to this deadly condition ultimately rests on the development of systemic therapy in targeting the molecular mechanisms underpinning the development of cerebral metastasis. Of all molecular targets identified to date, the cytoplasmic transcription factor STAT3 has a distinctive role in the development of targeted therapy that could regulate a multitude of prometastatic mechanisms. 

STAT3 is ubiquitously expressed in most tissues and constitutively activated in many tumor cells. In normal cells and under physiological conditions, the activation of STAT3 is rapid and transient and the effects of conditional ablation of STAT3 in adult tissues are mild [54]. These characteristics render STAT3 inhibition of limited toxic effects to normal cells and of significant impact in halting the progression of tumor metastasis. Currently numerous STAT3 small molecule inhibitors, some with penetration into the CNS, have been studied in various laboratories [51]. With solidified focus on STAT3 as a therapeutic target, further success in the development of an anti-STAT3 therapy may be close at hand, bringing new hope of preventing and treating this perilous condition. 
